# Sepsis-Like Systemic Inflammation Induced by Nano-Sized Extracellular Vesicles From Feces

**DOI:** 10.3389/fmicb.2018.01735

**Published:** 2018-08-07

**Authors:** Kyong-Su Park, Jaewook Lee, Changjin Lee, Hyun Taek Park, Jung-Wook Kim, Oh Youn Kim, Sae Rom Kim, Madeleine Rådinger, Hoe-Yune Jung, Jaesung Park, Jan Lötvall, Yong Song Gho

**Affiliations:** ^1^Department of Life Sciences, Pohang University of Science and Technology, Pohang, South Korea; ^2^Krefting Research Centre, Institute of Medicine, University of Gothenburg, Gothenburg, Sweden; ^3^R&D Center, NovMetaPharma Co. Ltd., Pohang, South Korea; ^4^Department of Mechanical Engineering, Pohang University of Science and Technology, Pohang, South Korea

**Keywords:** extracellular vesicles, exosomes, feces, membrane vesicles, outer membrane vesicles, sepsis-like systemic inflammation

## Abstract

Nano-sized extracellular vesicles (EVs), including exosomes, microvesicles, and other types of vesicles, are released by most mammalian cells and bacteria. We here ask whether feces contain EVs of mammalian and/or bacterial origin, and whether these EVs induce systemic inflammation. Fecal extracellular vesicles (fEVs) were isolated from mice and humans. The presence of EVs from Gram-negative and Gram-positive bacteria was detected by enzyme-linked immunosorbent assay using anti-lipid A and anti-lipoteichoic acid antibodies, whereas Western blot using anti-beta-actin antibody was employed to detect host-derived EVs in the fEVs. Further, fEVs were administered into mice by intraperitoneal injection, and inflammatory responses were investigated in the peritoneum, blood, and lungs. The role of TLR2 and TLR4 were studied using knockout mice. Significant quantities of EVs were present in feces from mice as well as humans, and derived from Gram-negative and Gram-positive bacteria, as well as the host. Bacteria-free fEVs introduced into the peritoneum induced local and systemic inflammation (including in the lungs), but fEVs from germ-free animals had weaker effects. This pronounced local and systemic inflammatory responses seemed to be induced by EVs from both Gram-negative and Gram-positive bacteria, and was attenuated in mice lacking TLR2 or TLR4. Our findings show that fEVs cause sepsis-like systemic inflammation, when introduced intraperitoneally, a process regulated by TLR2 and TLR4.

## Introduction

The intestinal tract contains vast quantities of bacteria, and is exposed continuously to environmental antigens and commensal microbes that normally live in a symbiotic and mutually beneficial relationship with their hosts (O’Hara and Shanahan, 2006; Artis, 2008; Hodges and Hecht, 2012). However, even a minute disruption of the barrier between the gut lumen and peritoneum can induce severe local and systemic inflammatory responses, such as peritonitis and sepsis, which are life-threatening conditions (Schein et al., 1997; Brook, 2003; Pieracci and Barie, 2007). When intestinal bacteria penetrate from the gut lumen into the peritoneum, peritonitis is induced. Overwhelming infection with intestinal bacteria will lead to excessive activation of the immune responses, generalized inflammation, hemodynamic instability, multiple organ dysfunction, and eventually death (Deitch, 1992; Pieracci and Barie, 2007).

However, not only whole bacteria, but also bacterial membrane components alone, have the ability to induce severe inflammatory responses in the mammalian hosts (Kuehn and Kesty, 2005; Lee et al., 2008; Tashiro et al., 2012; Kim J.H. et al., 2013; Park et al., 2013; Kim et al., 2015). Specifically, it has recently been shown that nano-sized extracellular vesicles (EVs) derived from Gram-negative bacteria, such as *Escherichia coli*, can provoke severe immune responses and signs of septic shock, without the presence of living bacteria (Park et al., 2010; Shah et al., 2012). This effect is substantially more potent than that of bacterial endotoxin lipopolysaccharide (LPS) alone, suggesting that the presence of other bacterial components strongly enhances the induced inflammation. It is also known that membrane components derived from Gram-positive bacteria can over-activate the immune responses by binding to pattern-recognition receptors (Moreillon and Majcherczyk, 2003; Hanson and Neely, 2012), and recently we reported that Gram-positive bacteria, such as *Staphylococcus aureus*, can also release EVs (Lee et al., 2009; Hong et al., 2011).

The aim of the current study is to determine whether EVs derived from feces can induce local and/or systemic inflammatory responses if introduced into the peritoneal cavity, and to which degree EVs from different types of bacteria contribute to such effects. Thus, EVs were isolated from feces of wild-type mice, germ-free mice, and humans (fecal extracellular vesicles; fEVs), and characterized in relation to bacterial or host sources. The isolated fEVs were introduced into the peritoneum, and inflammatory responses were determined locally, in the blood as well as in the lung, a distant organ. We also determined the degree of inflammatory responses by macrophages treated with fEVs and Gram-negative bacterial EV-removed fEVs. Finally, the relative contribution of TLR2 and TLR4 in the inflammatory responses to fEVs *in vivo* was studied using specific knockout (KO) mice.

## Materials and Methods

### Mice

This study was carried out in accordance with the recommendations of the Institutional Animal Care and Use Committee at Pohang University of Science and Technology, Pohang, South Korea. The protocol was approved by the Institutional Animal Care and Use Committee at Pohang University of Science and Technology (Approval number: 2011-01-0022). Wild-type and TLR4 KO of the C57BL/6 genetic background (6 weeks old) were purchased from the Jackson Laboratory (Bar Harbor, ME, United States). TLR2 KO mice and germ-free mice of the C57BL/6 genetic background (6 weeks old) were obtained from Dr. Myoung Ho Jang (Pohang University of Science and Technology), and Dr. Charles D. Surh (Pohang University of Science and Technology), respectively. Except the germ-free mice, mice were reared under specific pathogen-free conditions in the animal care facility of Pohang University of Science and Technology. The germ-free mice were raised in sterile flexible film isolators (Class Biologically Clean Ltd., Madison, WI, United States).

### Preparation of fEVs

Feces were collected from wild-type and germ-free mice or four healthy male volunteers (27–30 years old) daily at the same time, and stored at -80°C until use. Mouse feces were collected from the cage, and EVs derived from feces of germ-free mice were used as “blank” isolations to quantitate baseline of environmental EVs. For human fecal samples, as previously reported (Oshiki et al., 2018), all volunteers provided oral consent to provide the samples. All samples were anonymized and there was no possibility of personal identification. About 35 g of fecal materials were used for purification of fEVs for each group. To prepare fEVs, feces were dissolved with phosphate-buffered saline (PBS) at 4°C for 10 min by inverting. About 4 mL of PBS were used to dissolve every gram of feces, and no physical disruptions were needed to dissolve the feces in PBS. Insoluble materials and cell debris were removed by centrifugation at 800 × *g* for 5 min at 4°C for three times, and then centrifugation at 5,000 × *g* for 15 min at 4°C for three times. The supernatant was placed onto 1 mL of 0.8 M sucrose and 2.5 M sucrose in HEPES-buffered saline (HBS; 20 mM HEPES, 150 mM NaCl, pH 7.4), and then centrifuged at 100,000 × *g* for 2 h at 4°C, using an ultracentrifuge (Optima LE-80K; Beckman Coulter, Brea, CA, United States) with SW32Ti rotor. The interface layer between 0.8 and 2.5 M sucrose cushion was harvested and diluted 30-fold with PBS. The diluted solution was filtered through a 0.45 μm pore-sized filter, and the filtrate was placed onto 1 mL of 0.8 M sucrose and 2.5 M sucrose in HBS, and then centrifuged at 100,000 × *g* for 2 h at 4°C, using an ultracentrifuge (Optima LE-80K) with SW41Ti rotor. Finally, fEVs were prepared by taking the interface layer between 0.8 and 2.5 M sucrose cushion. The protein concentration of fEVs was quantified by Bradford assay (Bio-Rad Laboratories, Hercules, CA, United States). The fEVs derived from wild-type mice, germ-free mice, and humans were designated as ^Mouse^fEVs, ^GFMouse^fEVs, and ^Human^fEVs, respectively.

### Mammalian Cell Culture and Colon26 EV Preparation

Colon26 mouse colon adenocarcinoma (American Type Culture Collection, ATCC; Manassas, VA, United States) and RAW264.7 mouse macrophages (ATCC) were cultured in minimum essential medium (MEM; Gibco, Carlsbad, CA, United States) and Dulbecco’s modified Eagle medium (DMEM; Gibco), respectively. All culture media were supplemented with 10% fetal bovine serum (FBS; Gibco), 100 units/mL penicillin, and 0.1 mg/mL streptomycin. The cells were cultured at 37°C with 5% CO_2_ in a humidified incubator.

To prepare EVs derived from Colon26, Colon26 cells were rinsed twice with PBS, and incubated with serum-free MEM for 24 h. The conditioned medium was collected, and cell debris was removed by centrifugation at 500 × *g* for 10 min at 4°C, and then at 2,000 × *g* for 15 min at 4°C. The supernatant was pelleted by centrifugation at 100,000 × *g* for 2 h at 4°C, using an ultracentrifuge (Optima LE-80K) with Type45Ti rotor (Beckman Coulter, Brea, CA, United States). The pelleted Colon26 EVs were resuspended with PBS, and stored at -80°C until use.

### Bacterial Culture and Bacterial EV Preparation

*Escherichia coli* DH5α and *S. aureus* ATCC14458 were purchased from ATCC. EVs derived from *E. coli* DH5α and *S. aureus* ATCC14458 were prepared as previously described (Lee et al., 2007, 2009). *E. coli* DH5α and *S. aureus* ATCC14458 were cultured in 7.2 L of lysogeny broth (1% tryptone, 0.5% yeast extract, 1% NaCl, pH 7.0; Merck, Darmstadt, Germany) or nutrient broth (0.5% peptone, 0.3% meat extracts, pH 7.0; Merck), respectively. Bacteria were cultured at 37°C with gentle shaking (150 rpm), until A_600_ = 1.5. Bacterial cultures were centrifuged at 6,000 × *g* for 20 min at 4°C, and the supernatants were filtered through a 0.45 μm pore-sized vacuum filter. The filtrates were concentrated by ultrafiltration with QuixStand Benchtop System (GE Healthcare Life Sciences, Pittsburgh, PA, United States) equipped with a 100 kDa hollow-fiber membrane (GE Healthcare Life Sciences, Pittsburgh, PA, United States). The retentates were filtered through a 0.22 μm pore-sized vacuum filter, to remove any remaining cells. The resulting filtrates were pelleted by centrifugation at 150,000 × *g* for 3 h at 4°C, using an ultracentrifuge (Optima LE-80K) with Type45Ti rotor (Beckman Coulter, Brea, CA, United States), and resuspended with PBS. The protein concentrations of the prepared EVs were quantified with Bradford assay (Bio-Rad Laboratories, Hercules, CA, United States).

### Characterization of fEVs

To morphologically characterize the fEVs, the fEVs were subjected to transmission electron microscopy (TEM). The purified fEVs in PBS were placed on 400-mesh copper grids (Electron Microscopy Services, Hatfield, PA, United States). The fEV-absorbed grids were washed with deionizied water, and negatively stained with 2% uranyl acetate (Ted Pella, Redding, CA, United States). TEM images were acquired using JEM1011 electron microscope (JEOL, Tokyo, Japan) at an accelerating voltage of 100 kV. To determine the size distribution of fEVs, dynamic light scattering was performed with Zetasizer Nano ZS (Malvern Instruments, Worcestershire, United Kingdom) equipped with a 633 nm laser line, at the scattered intensity for 10 × 30 s. For this analysis, the fEVs were diluted to a total protein concentration of 5 μg/mL in 2 mL volume of PBS. The measurement was obtained five times with 1 min intervals at 25°C.

To investigate origins of fEVs, enzyme-linked immunosorbent assay (ELISA) and Western blot analyses were conducted. For ELISA, various amounts of fEVs and EVs derived from *E. coli* and *S. aureus* were coated on black opaque microtiter plates (Greiner Bio-One Ltd., Frickenhausen, Germany). After overnight incubation, the plates were blocked with 3% bovine serum albumin/PBS for 2 h. To detect EVs derived from Gram-negative and Gram-positive bacteria, anti-lipid A antibody (Abcam, Cambridge, MA, United States) or anti-lipoteichoic acid (LTA) antibody (Abcam, Cambridge, MA, United States) was treated for 2 h, and then horseradish peroxidase-conjugated secondary antibody was treated for 1 h. Relative chemiluminescence was measured using a chemiluminescent substrate (Roche Diagnostics GmbH, Mannheim, Germany). For Western blot, fEVs (20 μg in total protein amounts) and Colon26 EVs (30 μg in total protein amounts) were subjected to sodium dodecyl sulfate-polyacrylamide gel electrophoresis, and transferred to a polyvinylidene fluoride membrane. The membrane was blocked with 3% skim milk/PBS for 2 h. To detect host-derived EVs, anti-beta-actin antibody (Sigma-Aldrich, St. Louis, MO, United States) was treated for 2 h, and then horseradish peroxidase-conjugated secondary antibody was treated for 1 h. The immunoreactive bands were detected with a chemiluminescent substrate (iNtRON Biotechnology, Seongnam, South Korea).

### Protocol for fEV-Induced Inflammation in Mice

To induce inflammation in mice, fEVs from wild-type mice, germ-free mice, and humans (100 μg in total protein amounts) were intraperitoneally administered into wild-type, TLR2 KO, and TLR4 KO mice. At various time points (0, 3, 6, 12, and 24 h) after intraperitoneal administration of fEVs, the peritoneal lavage fluid, blood, and bronchoalveolar lavage (BAL) fluid were retrieved from the mice. As previously reported (Park et al., 2010; Kim O.Y. et al., 2013), the peritoneal lavage fluid and BAL fluid were harvested by instilling and aspirating two successive 1 mL of PBS to the peritoneal cavity and bronchus, respectively, and the blood was retrieved by cardiac puncture. The obtained samples were centrifuged at 600 × *g* for 10 min, then 1,500 × *g* for 10 min, and the supernatants were stored at -80°C until measuring cytokine concentrations. Tumor necrosis factor (TNF)-α and interleukin (IL)-6 in the peritoneal lavage fluid, serum, and BAL fluid were quantified by DuoSet ELISA kit (R&D Systems, Minneapolis, MN, United States). The pelleted cells in the peritoneal lavage fluid and BAL fluid were resuspended in 0.2 mL of PBS, and counted using the light microscopy. Differential count of cells in the peritoneal lavage fluid and BAL fluid were quantified by counting 300 cells using a light microscope after staining with Diff-Quik (Dade Behring, Marburg, Germany). Inflammatory cells were classified as following: macrophages, lymphocytes, neutrophils, and eosinophils.

### Whole Body Imaging

After incubation of ^Mouse^fEVs with Cy7-mono-NHS ester (5 μM, GE Healthcare Life Sciences, Pittsburgh, PA, United States) for 2 h at 37°C, Cy7-labeled ^Mouse^fEVs (^Mouse^fEVs_Cy7_) were isolated by centrifugation at 150,000 × *g* for 3 h at 4°C, as previously reported (Jang et al., 2015). Wild-type mice were intraperitoneally administered with ^Mouse^fEVs_Cy7_ (100 μg in total protein amounts). Before administration, as well as at 10 min, 3, 6, 12, and 24 h after administration, Cy7 fluorescence in the mice was measured by IVIS spectrum (Caliper Life Sciences, Hopkinton, MA, United States) at Pohang Center for Evaluation of Biomaterials, Pohang Technopark, Pohang, South Korea. At 3 and 24 h after administration, mice were sacrificed, and Cy7 fluorescence was quantified in organs including the liver, lung, spleen, kidney, heart, and small intestine. Living Image 3.1 software (Perkin Elmer, Waltham, MA, United States) was used to measure radiant efficiency, and the efficiency was normalized by tissue weights.

### Inhibition of TLR4 and Cytokine Induction by Macrophages

To remove LPS-positive EVs in ^Mouse^fEVs, ^Mouse^fEVs were applied to a polymyxin B column (Thermo Scientific, Rockford, IL, United States), according to the manufacturer’s instructions. The removal of LPS-positive EVs with retaining LTA-positive EVs in ^Mouse^fEVs were verified by ELISA using anti-lipid A antibody (Abcam, Cambridge, MA, United States) and anti-LTA antibody (Abcam, Cambridge, MA, United States).

RAW264.7 cells were seeded on a 24 well plate (5 × 10^4^ cells/well), and were pre-incubated with vehicles or TLR4 antagonist [1 μg/mL; LPS from *Rhodobacter sphaeroides* (InvivoGen, San Diego, CA, United States)] for 30 min at 37°C. Then, the cells were treated with ^Mouse^fEVs (5 μg/mL in total protein concentration) with 0.5% FBS for 15 h at 37°C. The conditioned media were harvested, and the concentrations of TNF-α and IL-6 were measured by DuoSet ELISA Development kit (R&D Systems).

### Immunohistochemistry

At 3 h after intraperitoneal administration of ^Mouse^fEVs (100 μg in total protein amounts) into wild-type, TLR2 KO, and TLR4 KO mice, the lungs were excised after whole body perfusion. The harvested lungs were fixed with 4% paraformaldehyde, embedded in paraffin, sectioned (4 μm thickness), and deparaffinized. The deparaffinized lungs were rehydrated by passage through xylene and a grade alcohol series. After antigen retrieval was performed by incubation in citrate buffer in a pressure cooker, the lung sections were blocked with 5% horse serum/Tris-buffered saline with 0.02% Triton X-100 for 2 h, and then treated with anti-Ly6C/G (Abcam, Cambridge, MA, United States) 1:100, anti-SP-C (Santa Cruz Biotechnology, Santa Cruz, CA, United States) 1:100, or anti-^Mouse^fEVs antibody (lab-made polyclonal antibody from rabbits) 1 μg/mL for overnight at 4°C. After treatment of AlexaFluor-conjugated secondary antibody for 2 h, the lung sections were counter-stained with Hoechst (Sigma-Aldrich, St. Louis, Missouri, United States). All images were acquired with an FV1000 Olympus confocal microscope (Olympus, Tokyo, Japan), and analyzed with FV10-ASW 3.0 software (Olympus, Tokyo, Japan). The numbers of neutrophils per field were measured by counting random fields in fluorescent images from each lung tissue.

### Statistical Analysis

The resulting values were expressed as mean and standard error mean (SEM), and Student’s *t*-test was used to find any significance. The level of statistical significance was higher than 95% confidence in all the analyses.

## Results

### Purification and Characterization of fEVs

**FIGURE 1:**
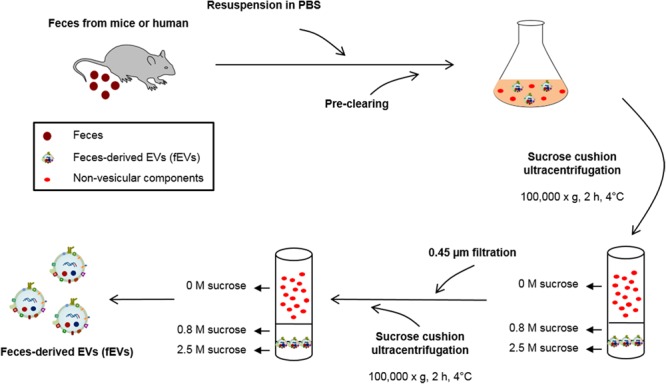
Schematic illustration of the purification methods of fecal extracellular vesicles (fEVs). The fEVs were isolated from feces of mice and humans as illustrated. PBS, phosphate-buffered saline.

**FIGURE 2:**
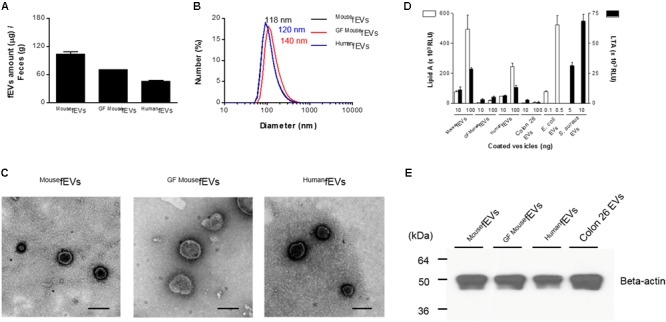
The fEVs originate from intestinal bacteria and the hosts. **(A)** The yields of fEVs measured in the total protein amounts (*n* = 3). **(B)** The size distribution of the fEVs measured by dynamic light scattering (*n* = 5). **(C)** TEM images of the purified fEVs. Scale bars = 200 nm. **(D)** The fEVs were analyzed for the content of lipid A (Gram-negative bacterial component) and LTA (Gram-positive bacterial component) by ELISA (RLU, relative luminescence unit) (*n* = 3). EVs derived from *E. coli, S. aureus*, and Colon26 were used as controls for Gram-negative and Gram-positive bacterial as well as host origins, respectively. **(E)** The fEVs (20 μg in total protein amounts) were analyzed by Western blot, using anti-beta-actin antibody, and Colon26 EVs (30 μg in total protein amounts) were used as controls for mouse origins. Error bars indicate SEM.

The fEVs were purified from feces of mice and humans, as illustrated in **Figure 1**. After dissolving the feces with PBS, insoluble materials and cell debris in feces were removed by serial centrifugations. The fEVs were finally purified by two rounds of sucrose cushion ultracentrifugation. The average amounts (in total protein amounts) of ^Mouse^fEVs, ^GFMouse^fEVs, and ^Human^fEVs were 103.7 ± 8.9 μg, 70.1 ± 1.4 μg, and 45.7 ± 3.6 μg were isolated from every gram of feces, respectively (**Figure 2A**). The average diameters of ^Mouse^fEVs, ^GFMouse^fEVs, and ^Human^fEVs were 118.1 ± 11.3 nm, 140.9 ± 14.2 nm, and 120.5 ± 6.1 nm, respectively, as determined through dynamic light scattering (**Figure 2B**). TEM revealed that the purified fEVs were spherical and appeared to have a closed membrane bilayer (**Figure 2C**).

We next investigated the origins of fEVs by identifying the marker molecules for prokaryotes and eukaryotes. Lipid A, the core component of Gram-negative bacterial endotoxin LPS, was identified from ^Mouse^fEVs, ^Human^fEVs, and *E. coli* EVs (**Figure 2D**). Moreover, LTA, a component of Gram-positive bacterial cell wall, was detected from ^Mouse^fEVs, ^Human^fEVs, and *S. aureus* EVs. However, ^GFMouse^fEVs and Colon26 EVs had only scarce amount of lipid A and LTA. Western blot detected beta-actin in all fEVs, as well as in Colon26 EVs (**Figure 2E**), showing that substantial amounts of fEVs are likely to originate from mammalian cells. Thus, the overall data suggest that fEVs originate from both Gram-negative and Gram-positive bacteria, as well as from their hosts.

### Peritoneal, Systemic, and Pulmonary Inflammation Induced by Intraperitoneal Administration of ^Mouse^fEVs and ^Human^fEVs, but Not by ^GFMouse^fEVs

**FIGURE 3:**
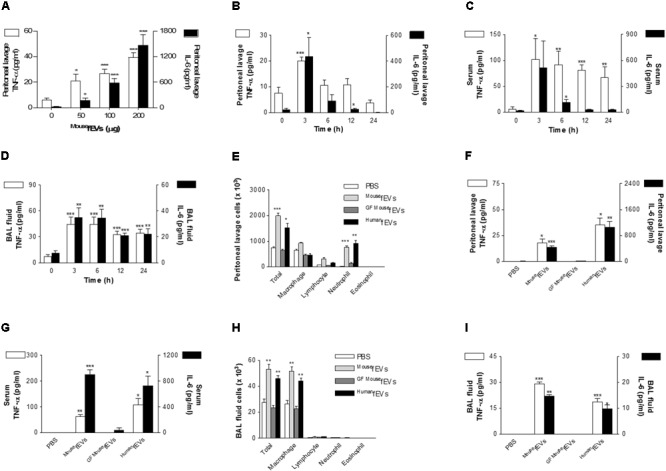
Comparison of peritoneal, systemic, and pulmonary inflammation by ^Mouse^fEVs, ^GFMouse^fEVs, and ^Human^fEVs. **(A)** Dose-dependent effects of ^Mouse^fEV-induced increase of the release of TNF-α and IL-6 in the peritoneal lavage fluid, after intraperitoneal introduction of ^Mouse^fEVs (*n* = 5). **(B–D)** The time course of ^Mouse^fEV-induced increase of the release of TNF-α and IL-6 in the peritoneal lavage fluid **(B)**, serum **(C)**, and BAL fluid **(D)**, after intraperitoneal introduction of ^Mouse^fEVs (100 μg in total protein amounts; *n* = 5). **(E–I)** At 3 h after intraperitoneal introduction of ^Mouse^fEVs, ^GFMouse^fEVs, or ^Human^fEVs (100 μg in total protein amounts; *n* = 5 for PBS and ^Mouse^fEVs group, *n* = 3 for ^GFMouse^fEVs group, and *n* = 5 for ^Human^fEVs group), the number of inflammatory cells **(E)**, and the concentration of TNF-α and IL-6 in the peritoneal lavage fluid **(F)** were quantified. The concentration of TNF-α and IL-6 in the serum **(G)**, the number of inflammatory cells **(H)**, and the concentration of TNF-α and IL-6 in the BAL fluid **(I)** were also measured. ^∗^*P* < 0.05, ^∗∗^*P* < 0.01, and ^∗∗∗^*P* < 0.001, respectively, when the data were compared to 0 h or PBS group. Error bars indicate SEM.

Various amounts of ^Mouse^fEVs were intraperitoneally administered to wild-type mice, resulting in the increase of the concentrations of TNF-α and IL-6 in the peritoneal lavage fluids in a dose-dependent manner (**Figure 3A**). As 100 μg ^Mouse^fEVs in protein was sufficient to induce the local inflammatory responses, we used 100 μg of fEVs in the subsequent analyses. Intraperitoneal administration of ^Mouse^fEVs to wild-type mice resulted in a substantial increase of the concentrations of TNF-α and IL-6 in the peritoneal lavage fluids (**Figure 3B**), serum (**Figure 3C**), and BAL fluid (**Figure 3D**), when compared to sham operation. The time-course of increases in the concentrations of TNF-α and IL-6 by ^Mouse^fEVs suggest that the local and systemic responses are occurring within hours and then subside, whereas the pulmonary response is significantly retained for 24 h.

Next, we compared local and systemic inflammatory effects of ^Mouse^fEVs with ^GFMouse^fEVs and ^Human^fEVs. Intraperitoneal administration of ^Mouse^fEVs and ^Human^fEVs resulted in a substantial increase in the peritoneal lavage fluid cells (**Figure 3E**), cytokines (**Figure 3F**), serum cytokines (**Figure 3G**), and BAL fluid cells (**Figure 3H**), and cytokines (**Figure 3I**), when compared to sham operation. In contrast, ^GFMouse^fEVs did not cause inflammation, suggesting that ^GFMouse^fEVs harbor few bacterial components, so they do not induce inflammation. Although the environmental contaminants can be contributed to the recovered fEVs, the contribution might be negligible in terms of proinflammatory effects of the fEVs, since ^GFMouse^fEVs showed little if any proinflammatory effects, when compared with ^Mouse^fEVs.

### Distribution of Intraperitoneally Administered ^Mouse^fEVs in Mice

**FIGURE 4:**
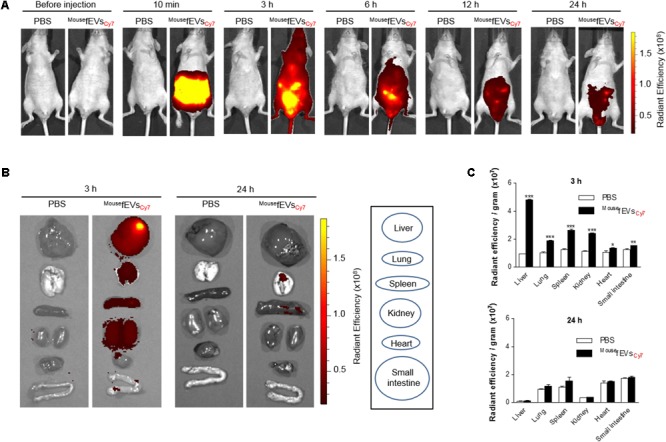
Distribution of intraperitoneally introduced ^Mouse^fEVs_Cy7_ in mice. The ^Mouse^fEVs_Cy7_ (100 μg in total protein amounts) were intraperitoneally introduced in mice. The Cy7 fluorescence of the whole body **(A)** or various tissues **(B)** was acquired by IVIS spectrum. Radiant efficiency was measured using Living Image 3.1 software, and normalized to tissue weight **(C)**. ^∗^*P* < 0.05, ^∗∗^*P* < 0.01, and ^∗∗∗^*P* < 0.001, respectively, when the data were compared to PBS group. Error bars indicate SEM (*n* = 3).

Wild-type mice were intraperitoneally administrated with ^Mouse^fEVs_Cy7_, to assess the distribution of ^Mouse^fEVs in mice. Whole body imaging showed strong accumulation of Cy7 fluorescence in the whole body at 3 h, and rare signals by 24 h (**Figure 4A**). The distribution of Cy7 fluorescence in different organs showed strong accumulation of Cy7 fluorescence in the liver, lung, spleen, kidney, and heart at 3 h, and then subsided (**Figures 4B,C**).

### Effects of TLR2 and TLR4 on the Responses to ^Mouse^fEVs

**FIGURE 5:**
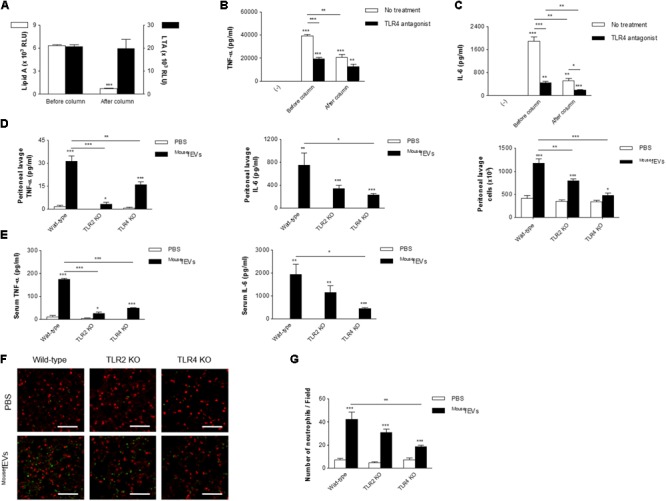
Both TLR2 and TLR4 contribute to systemic inflammation induced by ^Mouse^fEVs. **(A)** After passing ^Mouse^fEVs through a polymyxin B column, which binds LPS, ELISA using anti-lipid A and anti-LTA antibodies showed efficient removal of LPS-positive EVs (Gram-negative bacterial EVs) in ^Mouse^fEVs, but not of LTA-positive EVs (Gram-positive bacterial EVs) (*n* = 3). **(B,C)** RAW264.7 cells were pre-incubated with vehicles (open bars) or TLR4 antagonist (closed bar; LPS from *Rhodobacter sphaeroides*, 1 μg/mL) for 30 min, and treated with ^Mouse^fEVs (5 μg/mL in total protein concentrations), which either had or had not passed through the polymyxin B column, for 15 h, and the concentrations of TNF-α **(B)** and IL-6 **(C)** in the conditioned media were measured (*n* = 3). **(D,E)** Wild-type, TLR2 KO, TLR4 KO mice were sacrificed at 3 h after intraperitoneal introduction of ^Mouse^fEVs (100 μg in total protein amounts). The concentrations of TNF-α and IL-6 in the peritoneal lavage fluids **(D)** and serum **(E)** were measured (*n* = 5). The number of inflammatory cells in the peritoneal lavage fluids were also quantified **(D)**. **(F,G)** Wild-type, TLR2 KO, TLR4 KO mice were sacrificed at 3 h after intraperitoneal introduction of ^Mouse^fEVs (100 μg in total protein amounts). The lungs were excised after whole body perfusion and immediately fixed with paraformaldehyde, sectioned at 4 μm thickness, and subsequently stained with markers for neutrophils (anti-Ly6C/G antibody; green), and alveoli (anti-SP-C antibody; red). The lung images were acquired with a confocal microscope **(F)**. Scale bar = 100 μm. The numbers of Ly6C/G-positive cells per field were calculated **(G)** (*n* = 7). ^∗^*P* < 0.05, ^∗∗^*P* < 0.01, and ^∗∗∗^*P* < 0.001, respectively, when the data were compared to (-) or PBS group. Error bars indicate SEM.

To investigate the effect of depleting LPS-positive EVs from Gram-negative bacteria on the inflammatory activity of ^Mouse^fEVs, ^Mouse^fEVs were passed through a polymyxin B column. This approach significantly reduced the concentration of LPS-positive EVs from Gram-negative bacteria (**Figure 5A**). In contrast, the concentration of LTA-positive EVs from Gram-positive bacteria was not affected. Reduction of LPS-positive EVs from Gram-negative bacteria from ^Mouse^fEVs significantly reduced the release of TNF-α and IL-6 from RAW264.7 cells (**Figures 5B,C**; open bars). TLR4 antagonist significantly reduced both the release of TNF-α and IL-6 induced by ^Mouse^fEVs with or without having passed through the polymyxin B column (**Figures 5B,C**; closed bars), but the observed reduction was greater when ^Mouse^fEVs, which were not passed through the polymyxin B column, were used. Although not as high as ^Mouse^fEVs treatment, treatment of ^Mouse^fEVs having passed through the polymyxin B column with TLR4 antagonist was still able to induce the release of TNF-α and IL-6 by RAW264.7 cells (**Figures 5B,C**). All these observations suggest that both Gram-negative and Gram-positive bacterial EVs should be involved in ^Mouse^fEV-induced inflammation.

We next investigated the roles of TLR2 and TLR4 in ^Mouse^fEV-induced local and systemic inflammation. Intraperitoneal introduction of ^Mouse^fEVs to TLR2 KO mice significantly reduced the concentration of TNF-α in both the peritoneal lavage fluids (**Figure 5D**) and serum (**Figure 5E**), but did not significantly reduce the concentration of IL-6 in either compartment (**Figures 5D,E**), compared to wild-type mice. Intraperitoneal introduction of ^Mouse^fEVs to TLR4 KO mice significantly reduced the concentrations of TNF-α and IL-6 in both the peritoneal lavage fluids (**Figure 5D**) and serum (**Figure 5E**), compared to wild-type mice. However, intraperitoneal introduction of ^Mouse^fEVs to TLR2 KO and TLR4 KO mice significantly reduced the number of peritoneal lavage fluid cells, compared to wild-type mice (**Figure 5D**). In the lungs, significant sequestration of neutrophils was observed by the intraperitoneal introduction of ^Mouse^fEVs in wild-type mice (**Figures 5F,G**). A significant reduction in the sequestration of neutrophils induced by ^Mouse^fEVs was observed in TLR4 KO mice, but not in TLR2 KO mice compared to wild-type mice (**Figure 5G**). However, we were still able to observe the lung inflammation induced by ^Mouse^fEVs in both the TLR2 KO and TLR4 KO mice.

## Discussion

This study shows that feces contain significant amounts of fEVs, which are derived from both Gram-negative and Gram-positive bacteria, as well as from the host itself (most likely from intestinal epithelial cells). We further show that fEVs have the capacity to induce local inflammation, as well as systemic and pulmonary inflammation, when introduced in a sterile way into the peritoneum. The intraperitoneally introduced fEVs induced a neutrophilic peritoneal inflammation as well as sequestration of neutrophils into the lung tissues, which was further paralleled in a significant increase of airway macrophages. The cellular inflammation was closely associated with increased concentrations of TNF-α and IL-6, both locally and systemically. Our study further showed that the local and systemic inflammatory responses to fEVs in TLR2 KO and TLR4 KO mice were significantly reduced.

We showed that feces harbor EVs from both Gram-negative and Gram-positive bacteria, since the fEVs contain lipid A and LTA. Furthermore, a previous study reported the detection of CD63-positive EVs in feces from three children infected with rotavirus (Barreto et al., 2010), and our mouse study thus confirmed the presence of EVs containing a mammalian protein, beta-actin. Thus, fractions of the fEVs are likely to originate from the host, and most probably from the intestinal epithelium. In addition, considering that the gut microbiota is also constituted by a vast mycobiota (Huseyin et al., 2017), and that fungi also release EVs (Vallejo et al., 2012), fEVs could also be derived from gut fungi. Morphologically, many fEVs detected in this study were shown to be spherical and have a lipid bilayer. The sizes of fEVs may be related to the origins of EVs. As germ-free mice are devoid of fecal bacteria, ^GFMouse^fEVs do not contain bacterial EVs, which are relatively smaller than host cell-derived EVs. Thus, the average size of ^GFMouse^fEVs was bigger than those of ^Mouse^fEVs and ^Human^fEVs, which containing bacterial EVs.

Intraperitoneal introduction of the purified fEVs resulted in a strong inflammatory response in the peritoneum, as well as in the blood and the lungs. The local peritoneal inflammation, as well as the signs of systemic and pulmonary inflammation, was associated with a significant increase in the concentrations of TNF-α and IL-6 in all three compartments. We have previously shown that EVs from *E. coli* cultured *in vitro* could induce sepsis-like systemic inflammatory responses *in vivo* (Park et al., 2010), and the current study extend this finding by using feces, which represents multiple gut microbiota, as starting materials. Thus, this study suggests a putative pathogenic effect of EVs that may penetrate the gut mucosa to reach the peritoneum. In addition to our previous study with *E. coli* EVs, proinflammatory effects of different bacterial EVs have previously been described in EVs derived from *Pseudomonas aeruginosa* (Ellis et al., 2010; Park et al., 2013), *Salmonella enterica* serovar Typhimurium (Alaniz et al., 2007), *Neisseria meningitidis* (Bjerre et al., 2000), *Helicobacter pylori* (Ismail et al., 2003), *Moraxella catarrhalis* (Schaar et al., 2011), as well as *Mycobacterium tuberculosis* (Prados-Rosales et al., 2011). In most of these previous studies, the effects of the bacterial EVs have been studied either *in vitro*, or by intravenous or intranasal administration of the EVs. Thus, our current study is crucial, as it confirms that EVs of bacterial origins administrated to a closed body compartment could induce inflammation in a distant organ. Indeed, it has previously been suggested that EVs of bacterial origins deliver bacterial proteins at a distance (Bomberger et al., 2009), and it has been shown that sepsis patients have EVs in the circulation (Namork and Brandtzaeg, 2002). Furthermore, we have identified the presence of fEVs, or fragments of these EVs, in the lungs after intraperitoneal introduction. Although the small intestine seems like to be close from the peritoneum, fEVs are likely to be absorbed into the lymphatic vessels in the peritoneum (Mactier et al., 1987), so less amounts of fEVs may be distributed to the small intestine, when compared to other organs. Then, the absorbed fEVs may get into the heart via the lymphatic vessels and systemically distributed via the bloodstream. Like other nanoparticles, fEVs are likely to be retained for a long time in the organs with the developed reticuloendothelial systems, such as the liver, lungs, and spleen. Overall, these findings suggest that bacterial EVs that pass over the barrier function of the intestine, for example during infections or inflammatory diseases, could be distributed to other organs, and there cause an inflammatory response. Interestingly, the responses in the lungs to intraperitoneal introduction of fEVs resulted in an increase of macrophages in the BAL fluid, and an aggregation of neutrophils in the lung vasculature. It is not surprising that the neutrophils that gather in the lung vessels are not mirrored by neutrophilia in the BAL fluid, as it has previously been shown that neutrophils that have sequestered in the lungs are very slow to traffic into the lung tissues and bronchi (van Eeden et al., 2000). It has previously been shown that intestinal bacteria can be translocated to the extra-intestinal sites when the intestinal barrier ruptures in the patients undergoing abdominal surgery or trauma (Reddy et al., 2007). In such circumstances, bacteria or their components can evoke systemic inflammation, which leads to sepsis. We also used fEVs from humans to find meanings in the clinical circumstances. However, it would be valuable to track *in vivo* mouse distribution of human fEVs using Cy7-labeled human fEVs. Furthermore, the compositional characterization of fEVs may also be beneficial to further elucidate the mechanisms of inflammatory responses induced by fEVs.

Mice lacking either TLR2 or TLR4 had significant reduction in both the local and systemic inflammatory responses to intraperitoneally introduced fEVs. TLR2 responds to a greater extent to Gram-positive bacteria and LTA, whereas TLR4 responds to Gram-negative bacteria and LPS (Hennessy et al., 2010). Thus, the attenuated inflammatory responses in both TLR KO models suggest that EVs derived from both Gram-negative and Gram-positive bacteria contribute in the responses to fEVs, although it should be recognized that TLR2 can respond to some degrees to Gram-negative bacterial components. Interestingly, different degrees of the release of both TNF-α and IL-6 were observed in the different KO models, suggesting that each of these cytokines is regulated differently. Indeed, it has been suggested that Gram-negative and Gram-positive bacteria induce the release of TNF-α and IL-6 with different potencies (Hessle et al., 2005; Elson et al., 2007), and it has previously been suggested that TLR2 participate in the inflammatory responses against bacterial EVs (Prados-Rosales et al., 2011). However, to date, no *in vivo* studies have studied several pattern recognition receptors in the same study of bacterial EVs, which may help the relative contribution of these receptors in the inflammatory responses.

We have previously shown that introducing *E. coli* EVs intraperitoneally will lead to a sepsis-like death within 24 h (Park et al., 2010). In the current study, we used a similar concentration of EVs, but we did not observe the mortality seen in the previous study. This may be explained by the fact that fEVs contain lower concentration of *E. coli* EVs, which may be more potent than a mixture of EVs from feces. Furthermore, it is possible that bacterial EVs contribute significantly to the pathophysiology of systemic inflammatory response syndrome. Indeed, bacteremia is not often detected in sepsis (Munford, 2006), which may either be explained by insufficient culture techniques, or that the syndrome is induced by EVs that are systemically distributed from a local infection. Further studies using different approaches will be required to determine the relative contribution of EVs and whole bacteria in systemic inflammatory response syndrome.

This study for the first time shows that nano-sized EVs from feces have the capacity to induce local and systemic inflammation when introduced into the peritoneum. This pronounced inflammatory response seems to be induced by EVs from both Gram-negative and Gram-positive bacteria, and attenuated in both TLR2 and TLR4 KO mice. Thus, bacterial EVs in feces have strong proinflammatory properties, and may contribute to some of the pathology seen in sepsis.

## Author Contributions

K-SP, JL, CL, HP, J-WK, JLö, and YG conceived and designed the research. K-SP, JL, CL, HP, J-WK, OK, SK, and H-YJ performed the experiments. K-SP, JL, JP, JLö, and YG analyzed and interpreted the data. K-SP, JL, MR, JLö, and YG wrote the manuscript.

## Conflict of Interest Statement

H-YJ was employed by company NovMetaPharma Co. Ltd. The remaining authors declare that the research was conducted in the absence of any commercial or financial relationships that could be construed as a potential conflict of interest.
